# New concepts for generating interspecies chimeras using human pluripotent stem cells

**DOI:** 10.1007/s13238-021-00880-5

**Published:** 2021-10-11

**Authors:** Alejandro De Los Angeles, Jun Wu

**Affiliations:** 1 Department of Psychiatry, Yale University School of Medicine, New Haven, CT USA; 2 Department of Molecular Biology, University of Texas Southwestern Medical Center, Dallas, TX USA; 3 Hamon Center for Regenerative Science and Medicine, University of Texas Southwestern Medical Center, Dallas, TX USA

The worldwide paucity of transplantable organs for patients is a devastating problem. While different approaches have been conceived to address this donor organ shortage, one strategy that has garnered significant attention recently is the prospect of producing human organs in animals through a technique called interspecies blastocyst complementation based on the generation of interspecies chimeras. Interspecies blastocyst complementation works by injecting pluripotent stem (PS) cells from one species into organogenesis-disabled blastocysts of another species (Kobayashi et al., 2010). As the chimeric embryo develops, donor cells can fill the vacated developmental organ niche and enrich in a target organ of interest. In principle, interspecies blastocyst complementation may one day allow the generation of human organs inside animals. However, some ethical issues concerning research and potential human clinical use with chimeras persist (Hyun, 2019).

Despite the potential, however, successful interspecies organogenesis via blastocyst complementation has only been achieved between mice and rats (Kobayashi et al., 2010; Yamaguchi et al., 2017). To date, efforts to generate human organs and tissues in animals have been limited to endothelial cells and muscle, which can largely be attributable to limited human chimerism observed in several host species (Das et al., 2020; Maeng et al., 2021). The inefficient chimeric contribution of human PS cells to animal embryos likely derives from suboptimal PS cultures, evolutionary distance, and/or developmental incompatibility or xenogeneic barriers as the result of genomic evolution. Here, we summarize new advances in interspecies chimera research and recent efforts to define xenogeneic barriers (Fig. 1).

**Figure 1:**
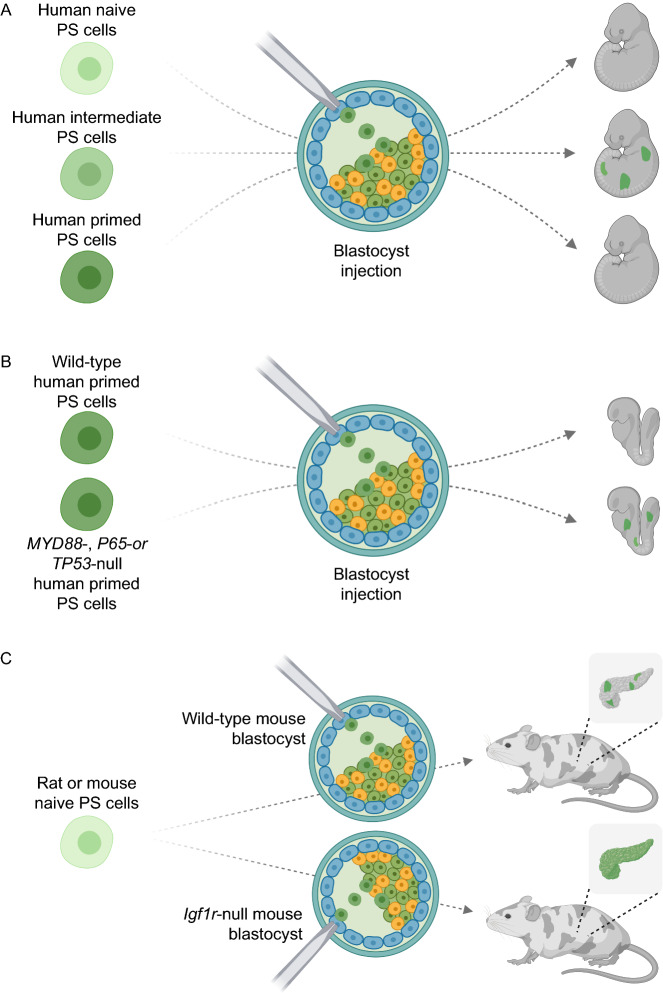
**Strategies to improve interspecies chimerism**. (A) Blastocyst chimera potential of human naïve, intermediate and primed PS cells. (B) Inhibition of cell competition via genetic perturbation of MYD88, P65 (also known as RELA), or TP53 promotes the survival and chimerism of human primed PS cells in mouse embryos. (C) Igf1r-null mouse embryos provide a “cell competitive” niche in multiple organs from mid-gestation for donor rat or mouse naïve PS cells to thrive. Created with BioRender.com

## NEW INTERSPECIES CHIMERA-COMPETENT PLURIPOTENT STEM CELLS

The generation of interspecies chimeras necessitate chimera competency of donor PS cells in the host species. However, only certain types of PS cells can form chimeras. Pluripotency in cultured PS cells spans across a spectrum of cellular states with distinct molecular and phenotypic features. At one end of the spectrum is naïve PS cells with relatedness to pre-implantation epiblast and capacity to form chimeras upon introduction to blastocysts. At the other end is primed PS cells that resemble peri-gastrulation epiblast and are capable of engrafting post-implantation epiblasts but not blastocysts. Therefore, the developmental stage of donor cells might be critical for chimera formation. Based on studies in rodents, the capacity to form blastocyst chimeras has been thought to be confined to naïve pluripotency, which in part underlies the great efforts spent in the past decade to generate human naïve PS cells. Surprisingly, however, human naïve PS cells reportedly showed little to no chimeric contribution in mice, pigs, and even rabbit and monkey embryos (Theunissen et al., 2016; Wu et al., 2017; Aksoy et al., 2021)

In contrast, human PS cells with features characteristic of a pluripotency state in between naïve and primed pluripotency demonstrated interspecies chimera competency, albeit at low levels (Wu et al., 2017). The same condition also supported derivation of horse PS cells, which contributed to chimera formation in E7 and E9 mouse embryos (Yu et al., 2020). Interestingly, several naïve-like human PS conditions were recently reported, which supported the generation of human-mouse chimeric embryos (Hu et al., 2020; Taei et al., 2020). Transcriptome analysis place some of these human naïve PS cells closer to E8-E10 than E6 human epiblast (Hu et al., 2020; Taei et al., 2020; Yu et al., 2020), suggesting they instead reside in intermediate rather than naïve pluripotency state. The need for independent validation notwithstanding, these new studies raise the intriguing possibility that human intermediate PS cells are better suited for interspecies blastocyst complementation experiments.

Of note, a recent study reported the identification of a human PS cell type competent for the generation of human-monkey *ex vivo* embryonic chimeras (Tan et al., 2021). Following the introduction of human extended pluripotent stem (EPS) cells into monkey embryos, the EPS cell derivatives survived, proliferated, and generated different peri- and early post-implantation cell types (Yang et al., 2017; Tan et al., 2021). Interestingly, a recent study analyzed the gene expression profile of mouse EPS cells and found that among different embryonic stages, stem cells grown in EPS cell conditions most closely corresponded to early post-implantation epiblast, a stage intermediate between naïve and primed pluripotency (Posfai et al., 2021). A previous analysis of human EPS cells showed relatedness to primed PS cells and transcriptional distance from pre-implantation embryo cells and human naïve cells (Stirparo et al., 2018). While more studies are needed, it is possible that human EPS cells also reside in an intermediate state of pluripotency. Nonetheless it remains unclear whether human EPS cells can generate interspecies chimeras when chimeric embryos are gestated in a uterus. It will be of great interest to assess the potential of human EPS cells to form interspecies chimeras gestated in utero, particularly with more distantly related livestock species such as pigs, the ideal animal host for organ generation.

These studies also raise new questions regarding human pluripotency. It will be of interest to further understand the nature of human intermediate PS cell states in relation to human embryonic states accessible through extended *ex vivo* culture of human embryos. Ultimately, our poor understanding of human pluripotency warrants continued research along this line, thereby enabling the development of new culture conditions that further improve the chimera-competency of human PS cells in animals.

## CELL COMPETITION IS AN IMPEDIMENT TO INTERSPECIES CHIMERA FORMATION

To date, attempts to generate human-animal chimeric embryos have resulted in notably lower levels of chimerism than intra- and inter-species chimeras between mice and rats, indicating that human cells were eliminated during development. However, whether human cell loss occurs via cell-autonomous and/or non-cell-autonomous process is unclear. Cell competition describes a fitness-sensing process during which “fitter” cells eliminate “loser” cells. Cell competition has been implicated in safeguarding normal development and maintaining tissue homeostasis. However, whether cell competition plays any role during interspecies chimera formation has not been studied until recently.

By co-culturing human and mouse PS cells, Zheng et al. uncovered a previously unrecognized mode of cell competition between species during which “loser” human PS cells were eliminated via apoptosis by “winner” mouse PS cells (Zheng et al., 2021). Intriguingly, competitive interaction between human and mouse cells was only observed in primed but not naïve pluripotency. In addition, primed PS cell competition appears to manifest in a more pronounced manner between distantly related (e.g., human and mouse) when compared with closely related (e.g., rat and mouse) species.

Through comparative RNA-seq analysis, the authors identified p53 and NF-κB signaling pathways, among others, were activated in co-cultured human cells. Genetic perturbation of these signaling pathways rescued the elimination of human PS cells by mouse PS cells. Researchers should exercise caution over extrapolating findings derived from cultured PS cells to developing embryos. Nonetheless, it appears that inhibition of these pathways improved the survival and chimerism of primed human PS cells in early mouse embryos.

It should be noted that the chimeric levels of primed human PS cells after disabling p53 or NF-κB signaling are likely still not enough to support interspecies organogenesis. In this regard, it’ll be interesting in future studies to investigate whether inhibiting cell competition would further enhance interspecies chimera competency of human intermediate PS cells that exhibited a higher baseline chimeric level than primed PS cells.

Importantly, this study also suggests it will be possible to exploit cell competition mechanisms to enhance interspecies chimerism in general. The authors observed *Tp53-* but not *MyD88-* deficient rat ES cells showed significantly improved chimerism in mouse embryos over wild-type rat ES cells, suggesting cell competition is also operative beyond primed pluripotency and is independent of Myd88/NF-κB signaling. It would be interesting to develop strategies to endow human cells with “super competitor” status to further improve interspecies chimerism.

## A CELL-COMPETITIVE NICHE COMPLEMENTATION STRATEGY

A greater level of chimeric contribution increases the probability that donor cells will contribute to target tissues. However, high degree of interspecies chimerism at early developmental stages often lead to embryonic lethality due to developmental incompatibly. Thus, it is imperative to find the middle ground between boosting overall chimerism and preserving embryo viability. Considering the inherent unpredictability of chimera experiments, it is difficult to gain precise control over chimeric levels during early development.

To overcome this, most recently Nishimura et al., reported a strategy to confer donor cells with a growth advantage over host cells at the fetal and postnatal stages (Nishimura et al., 2021). The authors generated a “cell competitive niche” in mouse embryos by globally knocking out insulin-like growth factor 1 receptor (Igf1r), thereby conferring donor wild-type mouse and rat PS cells with a growth advantage starting from mid-gestation. Interestingly, preferential donor cell growth was observed in several organs and tissues, raising the possibility for more complete and multi-organ complementation.

Notwithstanding the potential, more studies should be undertaken to resolve several outstanding issues. Whether the cell competitive niche strategy also entails cell competition or operates through cell-autonomous mechanism(s) is unclear. It will be of interest to determine whether using *IGF1R*-null host embryos facilitate generation of interspecies chimeras between distantly related species including humans and mice, and humans and pigs.

## CONCLUSION

Despite the huge promise for interspecies blastocyst complementation, there are some key technological and ethical barriers hindering broad adoption of this technology. There exist several hypotheses as to why it has been difficult to generate interspecies chimeras, including injected PS cell death, failure to differentiate, and large evolutionary differences between donor PS cells and host animal species. Additionally, divergence in ligand and receptor amino acid sequences, early post-implantation development, cell adhesion, and developmental speed represent important issues that reduce chimerism efficiency. Additionally, differences in the cell cycle and gestational length might play key roles in limiting interspecies chimera formation. One might surmise that interspecies chimera formation will require the synchronized development and differentiation of donor PS cell derivatives with the host animal embryo. Nonetheless, recent studies reporting the identification of human interspecies chimera-competent PS cells, the discovery of cell competition as an impediment to interspecies chimera formation, and development of a cell-competitive niche complementation strategy are facilitating the generation of PS cell-derived interspecies chimeras

Among the most controversial aspects of human interspecies chimera research is the fear of generating animals with human reproductive cells and intelligence (Hyun, 2019). Newly updated guidelines recommend strategies to address recent advances in stem cell research related to chimeric studies and several other applications (Hyun et al., 2021). Recently, several technological barriers limiting human cell chimerism in a growing animal embryo have been defined.

Since the first demonstration of generating rat pancreatic tissues in mice, much progress has been made in harnessing blastocyst complementation for interspecies organogenesis. Despite these advances, however, there is still a long way until the dream of generating human organs in animals for transplantation can be fulfilled. While strategies described in this comment, either independently or in tandem, may help us produce the first proof of concept of generating human fetal tissues in animals, there are probably other barriers at play, and it will be a long journey ahead before dream of growing transplantable human organs in animals is realized. Looking ahead, dedicated efforts to identify the molecular and cellular mechanisms underlying xenogeneic barriers will be needed. More importantly, it is imperative for scientists and bioethicists to engage public discussion and debate, to raise the awareness of the benefits of chimera research.
